# Eco-friendly EPDM nanocomposites reinforced with sugarcane bagasse-derived cellulose nanocrystals

**DOI:** 10.1186/s13065-025-01601-3

**Published:** 2025-08-13

**Authors:** Hebat-Allah S. Tohamy, Adel Koriem, Doaa E. El-Nashar

**Affiliations:** 1 Cellulose and Paper Department, National Research Centre, 33 El Bohouth Str, P.O. 12622, Dokki Giza, Egypt; 2 Polymers and Pigments Department, National Research Centre, 33 El Bohouth Str, P.O. 12622, Cairo, Dokki Giza, Egypt

**Keywords:** Sugarcane bagasse, Agricultural wastes, Cellulose nanocrystals (CNC), Ethylene propylene diene monomer (EPDM) rubber, Green nanocomposites, Sustaionable materials

## Abstract

The goal of this work is to prevent environmental pollution resulted from burned agriculture waste, offering a pathway with the potential to reduce CO₂ emissions compared to direct combustion of sugarcane bagasse. Cellulose nanocrystals (CNC) are produced by acid hydrolyzing cellulose obtained from sugarcane bagasse. It serves as a reinforcing filler within the ethylene propylene diene monomer (EPDM) rubber matrix. Transmission electron microscopy (TEM), thermogravimetric analysis (TGA), and X-ray diffraction (XRD) were employed to examine morphology, thermal properties and crystallinity respectively. The empirical crystallinity (LOI) and average hydrogen bond strength (MHBS) were determined using Fourier transform infrared spectroscopy (FTIR) for cellulose and cellulose nanocrystals (CNC). It was discovered that (CNC) is lower than CNC for both LOI and MHBS, demonstrating the breakdown of cellulose into CNCs. Two roll-mill was used to prepare EPDM/cellulose nanocrystals (CNC) nanocomposites. Two to ten parts of CNC per hundred rubbers were used. Curing characteristics, mechanical testing, thermogravimetric analysis (TGA), equilibrium swelling, and scanning electron microscopy (SEM) were used to assess the EPDM/(CNC) nanocomposites. SEM pictures, show that some clumping, particularly at higher percentages of (CNC), and a uniform dispersion of (CNC) at 8 phr. It has been demonstrated that when the loading of CNCs increases, the cure time lowers and cure rate index increased, simulating a shorter industrial production cycle. In comparison to EPDM at 8 phr (CNC) loading, Additionally, the results showed an increase in tensile strength from 2.68 to 10.82 MPa i.e. increased by 403.7% and increasing in elongation at break by 233.47%. Also the hardness increased to about 83.33%. The modulus at 50, 100, and 200%, as well as a decrease in equilibrium swelling that confirm the mechanical testing. So, the prepared (CNC) may be enhanced the mechanical properties of the evaluated nanocomposites comparing to EPDM vulcanizates free.

## Highlights

Bagasse from sugarcane is an agro industrial waste with a lot of products.

Cellulose nanocrystals is made from the bagasse of sugarcane to produce Sustaionable materials.

Mix cellulose nanocrystals and ingredients to EPDM to produce green nanocomposites.

Cellulose nanocrystals serve as effective filler that reinforces EPDM.

Mechanical properties of the nanocomposites were improved.

## Graphical Abstract

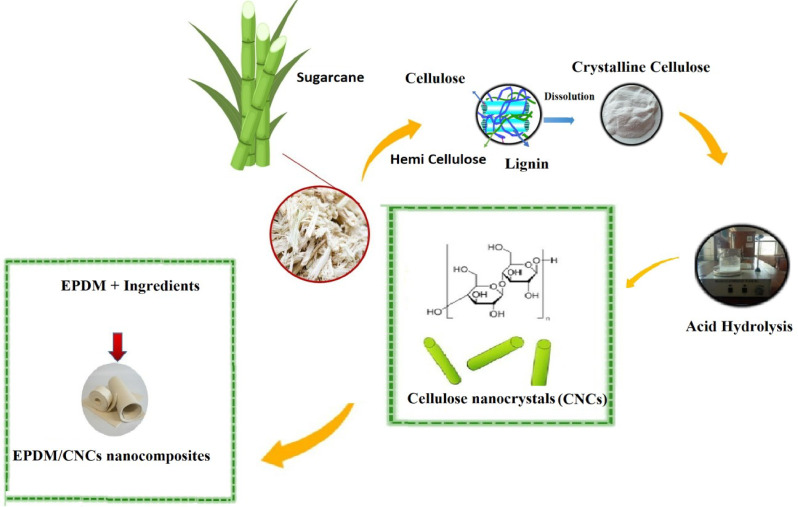

## Introduction

There are two types of rubber: synthetic and natural. Unlike synthetic rubbers like Styrene Butadiene Rubber (SBR), Ethylene Propylene Diene Monomer (EPDM), and Acrylonitrile Butadiene Rubber (NBR), tree latex contains natural rubber. Due to its inherent unique properties, EPDM, one of the most widely used “specialty” elastomers, was the perfect material to use for making nanocomposites containing cellulose nanocrystals (CNC) [1]. The saturated structure in the polymer backbone of EPDM causes ozonation, oxidation, weathering, and aging. In addition to its exceptional electrical and mechanical qualities, EPDM rubber has garnered a lot of interest from the industry because of its strong resistance to aging, heat, polar solvents, and other chemicals. The high levels of reinforcing agents and plasticizers that EPDM rubber accepts make processing it easy. By adhering to the circular economy’s guidelines, the development and production of innovative polymer composites of EPDM broadens the range of eco-polymer materials and advances current technology [2, 3].

Ethylene propylene diene monomer is one of the best synthetic rubbers, particularly for applications involving high temperatures and outdoor conditions [4, 5]. But generally, rubbers are employed as fillers and some compounding chemicals [6]. Fillers are used to enhance mechanical strength, processing characteristics, and reduce material costs. Fillers have given remarkable qualities like excellent mechanical qualities, thermal stability, longevity, and resistance to swelling, among many others [7]. Green fillers are therefore attracting the attention of researchers [8–11]. The unique qualities of cellulose nanocrystals (CNC) make them suitable for usage as a green filler. Cellulose is a renewable and biodegradable material that is most prevalent in nature. According to published research, cellulose nanocrystals (CNC) are considered promising green filler in the rubber industry and show significant performance enhancement because they come from natural, renewable sources such wood straw, biomass, pine trees, and many more [12–15]. (CNC) has qualities that make it appropriate for a variety of applications, such as enhanced crystallinity, low aspect ratio, strong mechanical properties [16–18], and a large surface area. Incorporating (CNC) as a filler in the polymer matrix results in improved mechanical characteristics, good dispersion, strength, and stiffness because hydrogen bonds and OH groups facilitate physical and chemical interactions between the (CNC) and the matrix due to their ability to disperse well in the matrix and their addition at the nanoscale, (CNC) are used as a filler to produce green nano-composites [19, 20]. They are prepared by in situ polymerization, solution intercalation, and melt intercalation [21–26].

While various sources for CNCs exist, this research focuses on the novel and sustainable utilization of sugarcane bagasse, an abundant agricultural waste product [27–29]. The majority of bagasse wastes are either burned or used to comfort animals. In light of the sugarcane industry projected growth and the need to promote sustainable development worldwide, numerous studies have been carried out to attempt and identify new applications for sugarcane bagasse residue. These investigations demonstrate how these residues can be used in a variety of ways as ashes. To create composite materials, sugar bagasse (SB) fibers have great mechanical strength could be included into polymeric matrices [30]. So we can use sugar bagasse as novel filler for EPDM rubber to improve the mechanical properties and reduce the environmental pollution.

Most people agree that lignocellulosic biomass is one of the best renewable resources on the planet [31]. To eliminate color and heavy metals from tainted water, electrospun nanofibers derived from agricultural cellulosic biomaterial wastes are utilized [32, 33].

Impacts of (CNC) surface chemical groups on the mechanical characteristics and vulcanization of natural rubber/cellulose nanocrystal nanocomposites, while the characteristics of nanofibrillated cellulose include excellent transparency, a large specific surface area, high hydrophilicity, high modulus and high strength. Research has indicated that its use as an additive in material modification can result in composites with high moduli of elasticity and strength [34–36].

The most prevalent naturally occurring polymer, cellulose has several special qualities, including being readily available, renewable, lightweight, and environmentally friendly as green filler. Cellulose has been effectively selected as a non-petroleum based reinforcing filler in rubber technology for the past ten years. Cellulose is known to have a strong hydrophilic nature. Therefore, the design for the ideal contact between the hydrophilic cellulose filler and the hydrophobic rubber matrix. This is the reason why designing sustainable rubber composites with cellulose filling is challenging [37–39].

In order to design EPDM-CNC nanocomposites with better qualities, this research study attempts to synthesis the (CNC) and optimizes its loading in EPDM. This study specifically explores the use of CNCs derived from sugarcane bagasse, highlighting a sustainable approach to waste valorization and a potential alternative to traditional CNC sources. The purpose of our work was novelty of using (CNC) as green filler EPDM to enhance the prepared EPDM nanocomposites, mechanical and thermal stability, reducing cost and reduction of environmental pollution. To evaluate the EPDM/ (CNC) nanocomposites shape, curing capabilities, thermal stability, and mechanical attributes, a variety of characterization techniques will be used.

## Materials

- EPDM (ethylene propylene diene monomer) was manufactured by Esso Chemi, Germany with ethylene content of 55% and density of 0.86 g/cm^3^.
- N-cyclohexyl-2-benzothiazole sulfenamide (CBS) was used as an accelerator with specific gravity of 1.27–1.31 g/cm^3^ at room temperature (25 ± 1 °C).
- Zinc oxide and stearic acid were employed as activators with specific gravity at 15 °C of 5.55–5.61 g/cm^3^ and 0.90–0.97 g/cm^3^, respectively.
- Naphthenic oil, deep green viscous oil, with specific gravity of 0.94–0.96 at 15 °C and viscosity of 80–90 poise at 100 °C was used and purchased from Sigma-Aldrich Company.
- Tetramethyl thiuram disulphide (TMTD), an odourless powder, with specific gravity 1.29–1.31, melting point 148.5^o^ C were used as accelerators.
- The elemental sulfur as vulcanizing agent as analytical grade that was applied as a fine, pale-yellow powder with a specific gravity of 2.04–2.06 at ambient temperature purchased from Sigma Aldrich.
- Trimethyl-1,2dihydroquinoline (TMQ) polymerization was employed as an antioxidant provided from Sigma Aldrich.

All reagents were of the analytical grade and distilled water was used throughout the experiments.

### Preparation of cellulose nanocrystals (CNC)

Bleached bagasse pulp was hydrolyzed with sulfuric acid by mixing 200 milliliters of 64 weight% sulfuric acid with 20 g of pulp, then heating the combination to 45 °C to produce (CNC).

The suspension was dialyzed against distilled water for five days, centrifuged, repeatedly cleaned with distilled water to neutralize the acid, and then ice water was added to stop the reaction. After being sonicated for 20 min, the resulting CNC was freeze-dried at -80 °C using a lyophillizer. For later usage, the CNC was preserved in powdered form [40]. The yield (%) of CNCs from bagasse to assess process efficiency is 60%.

The advantages of this method being, based on sulfuric acid hydrolysis, is a well-established and effective technique for producing CNCs with the desired properties [40]. When considering industrial-scale application, the fundamental steps of acid hydrolysis, neutralization, washing, and sonication are inherently scalable. Large-volume reactors could replace smaller laboratory equipment, and continuous flow systems could be implemented for enhanced efficiency. A significant challenge at industrial scale is managing the large volume of dilute sulfuric acid generated after the hydrolysis and washing steps. Directly discharging this acidic wastewater is neither environmentally sound nor economically viable. To address this, we would implement a multi-stage acid recovery system. Ion exchange resins could be employed to selectively remove impurities, ensuring cleaner acid recovery for various industrial applications. The specific recovery method would be chosen based on the desired purity for reuse, the economic feasibility, and the energy balance of the process.

### Rubber formulations and composites

In EPDM nano composites, the following mixing sequence was the most effective: In order to masticate the rubber and make it easier to combine with the additional ingredients, it was initially passed twice without banding at a roll opening of roughly 0.2 mm. It was then put into a laboratory two-roll mill with an outer diameter of 470 mm and a working distance of 300 mm, passing through a mill opening of roughly 1.5 mm. The friction gear ratio was set at 1.4:1 for 1.5 min while the slow roll was operated at 24 r min^− 1^. It takes around 3.5 min to add and mix (CNC) sequentially in situ using the masticated EPDM produced surrounding the roll mill. This was followed by the addition of the other precisely weighed additives, which included activators, processing oil, accelerators, antioxidants, and sulfur vulcanizing agent, in a standard order with an 8–10 min mixing period. As per ASTM D-3182 guidelines, Three to four cuts were made alternately from each side of the rolls since their temperature was kept at about 60 °C. About 13 to 15 min were spent combining everything. Before being molded, the resulting rubber mixtures were sheeted to create sheets that were at least 6 h old and had a thickness of 1–2 mm. ultimately, following mixing; the samples weights were verified. The EPDM mixes for the nanocomposites were compression molded (vulcanized) at 152 *±* 1^o^ C and 4 MPa of pressure in an electrically heated hydraulic press (Mackey Bowley, C1136199). The rheometer was used to determine the optimal time, which was 152 ± 1 °C and about 4 MPa of pressure, in compliance with ASTM D-1928. All of the ingredients are listed in the combinations in Table 1.

**Table 1:** Formulations of EPDM nanocomposites

Ingredients	Role	Content (phr)^a^					
		E0	E1	E2	E3	E4	E5
EPDM rubber Stearic acid Zinc oxide TMTD^b^ Sulfur CBS^c^ TMQ^d^ Naphthenic oil cellulose nanocrystals (CNC)	Rubber Activators Activators Accelerators Curing agent Accelerators Antioxidant Plasticizer Filler	100 1 3 0.5 2 1 1 3 0	100 1 3 0.5 2 1 1 3 2	100 1 3 0.5 2 1 1 3 4	100 1 3 0.5 2 1 1 3 6	100 1 3 0.5 2 1 1 3 8	100 1 3 0.5 2 1 1 3 10

^a^ Part per hundred parts of rubber. ^b^ Tetramethylthiuram disulfide. ^c^ N-cyclohexyl-2-benzothiazole sulfonamide. ^d^ Polymerized 2,2,4-trimethyl-1,2dihydroquinoline

### Characterization and measurements

#### Fourier transforms infrared spectra (FTIR)

FTIR analysis was carried out on a Mattson 5000 spectrometer (Unicam, UK) and the KBr disk method. Equations (1) and (2) were then used to determine the (LOI) empirical crystallinity and the (MHBS) average hydrogen bond strength.

1 $${\rm{MHBS = }}{{{{\rm{A}}_{{\rm{OH}}}}} \over {{{\rm{A}}_{{\rm{CH}}}}}}$$

2 $${\rm{LOI = }}{{{\rm{A1425}}} \over {{\rm{A900}}}}$$

A1425, A900, AOH, and ACH stand for the FTIR absorbance of the OH, CH, 1425, and 900 peaks, respectively [41].

#### The X-ray diffractions (XRD)

To investigate the crystallinity on X-ray powder diffraction, the Bruker D-8 Advance X-ray diffractometer (Germany) was utilized to analyze the diffraction patterns using copper (Kα) radiation (1.5406 Å) with a 40 kV voltage and 40 mA current. The 𝑑-spacing (thickness), crystallite size, dislocation density, and microstain can all be calculated using Eq. (3) [42].

3 $$Cr.I \left( \% \right) = {\rm{Sc}}/St X 100$$

Where Sc represents the area of the crystalline domain, St is the area of the whole domain, and Cr.I is the crystallinitr (%) each.

#### Electron microscope for transmission (TEM)

TEM pictures were taken using a JEM-2100 F (JEOL, Japan) equipped with a 200 kV accelerating voltage. A copper grid covered with carbon was spread out to allow the ethanol solution to evaporate after one millilitre of ethanol and a few drops of particle solution were combined.

#### Rheological characteristics

The Moving Die Rheometer (MDR 1), USA, is a TA device. Rheometric characteristics were assessed using ASTM D2084 at 152 ± 1 °C for scorch times (ts2, min), optimum cure time (tc90, min), minimum torque (ML), maximum torque (MH), and cure rate indices (CRI, min^− 1^).

#### The SEM (scanning electron microscope)

A scanning electron microscope (SEM) is a useful instrument for examining sample surfaces. By utilizing a Quanta FEG-250 to take SEM pictures of the samples, the surface morphology of the finished EPDM compounds was investigated. Over the period of six minutes, a very tiny layer of gold, five to ten nanometers thick, was applied to the samples. To provide a representative sample of the specimen’s general shape, the centre cross-section was chosen.

#### Analysis of thermogravimetric data (TGA)

A TGA Q500 (TA Instruments, USA) was used. Thermogravimetric analysis (TGA, USA) was assessed. It was employed to assess the thermal stability of vulcanized rubber nanocomposite films and powder filler with 0.1 mg thermobalance sensitivity. The samples were subjected to nitrogen gas at a flow rate of 50 mL per minute while being heated from 25 *±* ^o^C to 500 °C at a rate of 10 °C per minute. At the appropriate temperature, the samples weight loss was calculated using the differential curve.

#### Mechanical characteristics

Ethylene propylene diene monomer rubber sheets cut with an ASTM Wallace die cutter have physical-mechanical characteristics. Using the vulcanizates from the molded sheets, dumbbell-shaped specimens were created. The results are based on the average of five different dumbbell-shaped specimens with an estimated thickness of 1.5 mm and a working portion of 15 mm, and a consistent 4 mm width. A common thickness gauge was used to measure each specimen thickness. Rubber nanocomposite vulcanizates were tested for physicomechanical properties by using Zwick/Roell Z010 tensile tester machine with a load cell (Type: X force P and Nominal force: 10 KN) was used to measure the tensile strength (MPa), elongation at break (%), and modulus at 50, 100, and 200%, accordance with ASTM D412. Five replicates of the physicomechanical data were measured for the average. To test for hardness in compliance with ASTM D2240, a Durometer Shore A (Bareiss, Oberdischingen, Germany) was utilized.

#### Equilibrium swelling (Q)

The equilibrium swelling procedure was followed in accordance with D471-97.The specimens were immersed in toluene for 24 h at room temperature (25 *±* 1 °C) in order to induce the swelling. The apparent percentage change in mass was calculated, the percentage of swelling was calculated according Eq. (4).

4 $${\rm{Q\% = }}{{{\rm{[(W - W{^{\prime}})]}}} \over {{\rm{W{^{\prime}}}}}}{\rm{ \times 100}}$$

If w is the sample initial weight, then w` is the sample ultimate weight after swelling.

### Determination of the molecular weight between two crosslinks (Mc)

The crosslinking density (ν), mol/cm^3^ of EPDM vulcanizates was determined on the basis of solvent swelling measurements (toluene solvent for 24 h at 25 *+* 1 °C) using the Flory– Rehner equationin 5&6 [43].

5 $${{v = }}{{\rm{1}} \over {{\rm{2Mc}}}}$$

Where: Mc, g/mol is the molecular weight between crosslinks (g/mol).

6 $${\rm{Mc = }}{{{\rm{ - \rho VsV}}{{\rm{r}}^{{{\rm{1}} \over {\rm{3}}}}}} \over {{\rm{[ln(1 - Vr) + Vr + \chi V}}{{\rm{r}}^{\rm{2}}}{\rm{]}}}}$$

where: ρ is the density of rubbers (ρ EPDM is 0.86 g/cm^3^ ), versus is the molar volume of the solvent (toluene) = 106.35 cm^3^ /mol, χ is the interaction parameter of EPDM rubber, that is 0.49 and Vr is the volume fraction of swollen rubber that can be obtained from the mass and density of rubber samples and the solvent (Vr = 1/ 1 + Q).

## Results and discussion

The characterization of the prepared CNC was carried out using FTIR, XRD, SEM as well as the particle size was measured by TEM [44–46]. Also, the thermal gravimetrical analysis (TGA) was investigated. The CNC nanomaterial and the other ingredients were incorporated with EPDM matrix to obtain green novel nanocomposites. The evaluation of the prepared nanocomposites was done using the Moving Die Rheometer, SEM, TGA, also measuring mechanical and equilibrium swelling properties.

### Transmission electron microscope (TEM)

**Fig. 1:**
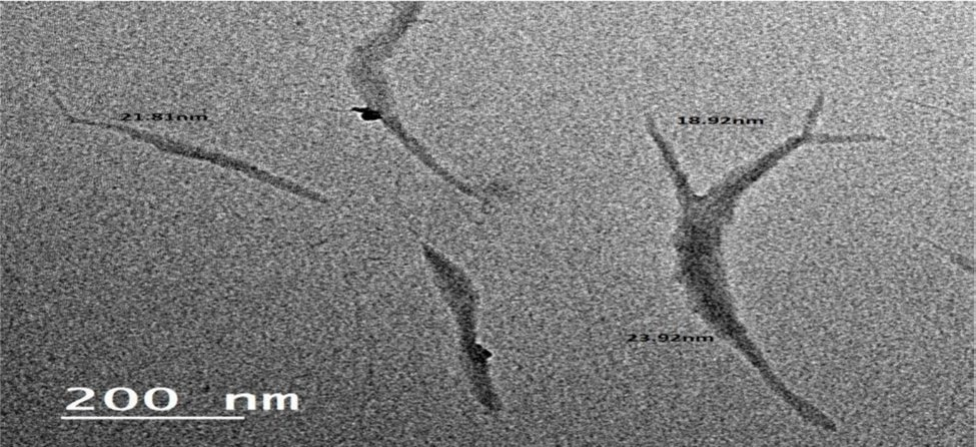
TEM image with

### Magnification 1000X of cellulose nanocrystals (CNC)

The CNC suspensions, TEM micrograph was assessed using TEM. As shown in Fig. 1, the fibers in the final suspension had an aspect ratio 12.21 nm and a diameter of nanoscale. The picture showed long fibrils that ranged in diameter from 18.92 to 23.69 nm. Most of the fibers had an aspect ratio.

### Fourier transform infrared spectroscopy (FTIR)

**Fig. 2:**
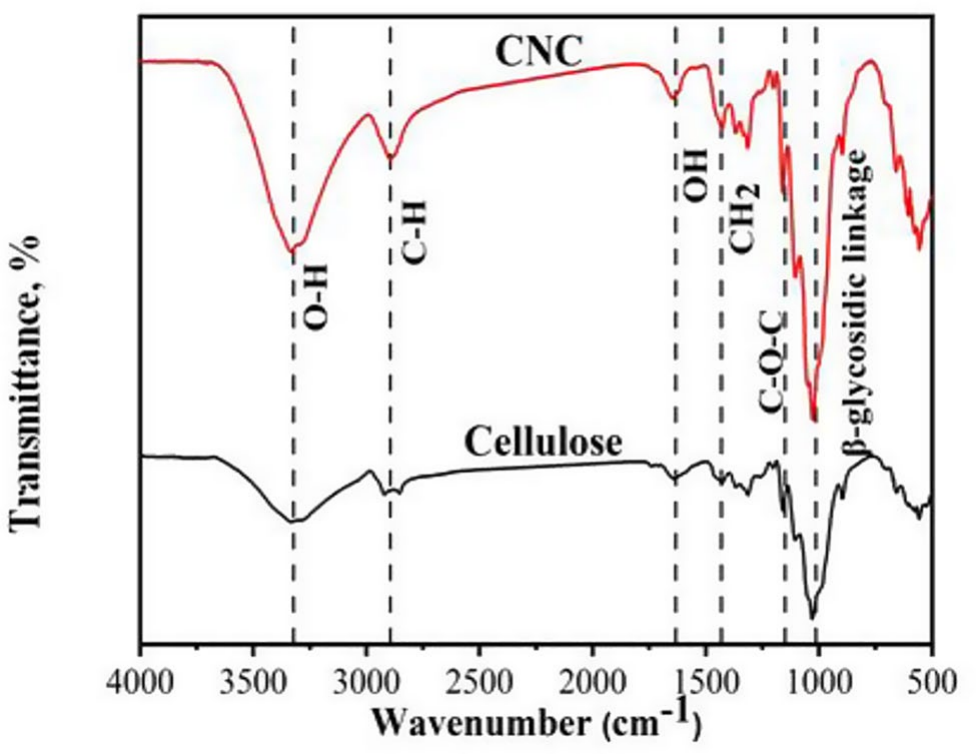
FTIR of cellulose and cellulose nanocrystals (CNC)

Figure 2 shows the FT-IR spectra of cellulose and (CNC). The characteristic bands are located between 3326 and 3335 cm^− 1^ for (O–H), 2895 and 2929 cm^− 1^ for (C–H), 1643 and 1650 cm^− 1^ (OH bending of adsorbed water), 1429 and 1432 cm^− 1^ (CH2 bending), 1162 and 1163 cm^− 1^ (C–O–C of pyranose ring), and 1031 and 1033 cm^− 1^ (β-glycosidic linkage between glucose units in cellulose) [34]. The OH peak was shifted from the lower value 3326 cm^− 1^ for cellulose to the higher value 3335 cm^− 1^ for CNCs which meaning low H-bond strength. The calculated LOI and MHBS of cellulose are lower than CNC which proved the breakdown of cellulose into CNCs (Table 2). The LOI and MHBS values are agree with the decreasing of H-bonding after the preparation of CNC.

**Table 2:** MHBS and LOI of cellulose and cellulose nanocrystals (CNC)

Sample	LOI$\:\:\frac{\varvec{A}1425}{\varvec{A}900}$	MHBS $\:\frac{\varvec{A}\varvec{O}\varvec{H}}{\varvec{A}\varvec{C}\varvec{H}}$
**Cellulose**	4.39	2.15
**Cellulose nanocrystals (CNC)**	1.43	0.89

### X-ray diffraction

**Fig. 3:**
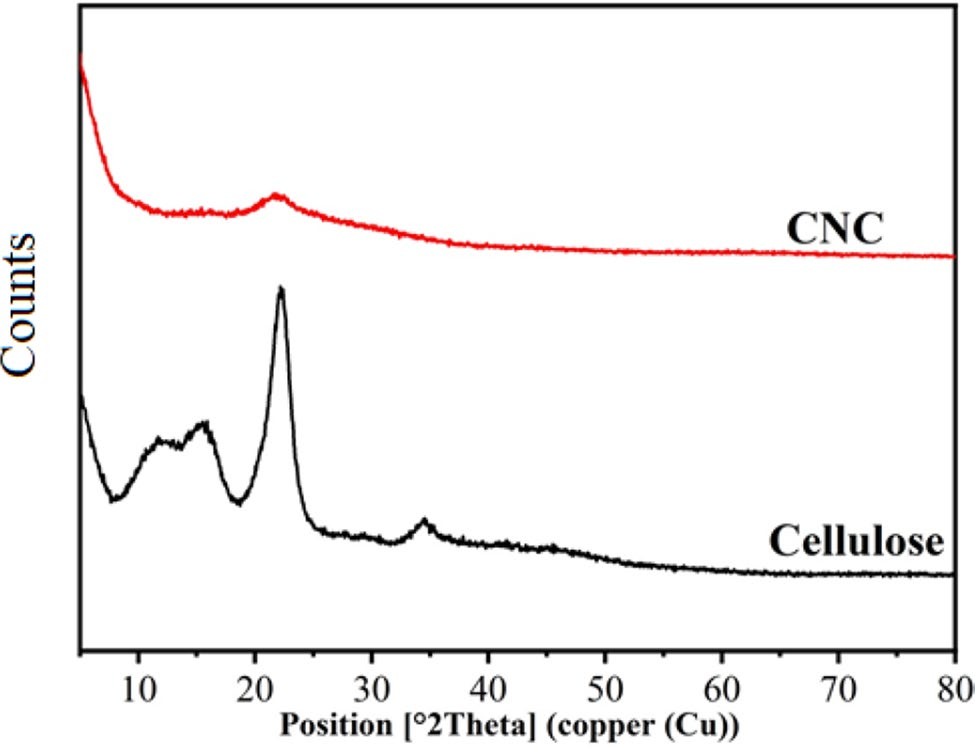
XRD analysis of cellulose and cellulose nanocrystals (CNC)

The 11.89, 15.88, and 22.17° peaks are the distinctive diffraction peaks of cellulose. The division in the reflection between 15.88 and 22.17° indicates the production of cellulose II as shown in Fig. 3 [41]. One peak was visible in the (CNC) at 21.78°.

Table 3 displays the values of Cr.I (%) and d spacing (nm). The computed crystallinity Cr.I (%) for cellulose and (CNC) was 61.02 and 55.55%, respectively. This indicates that the cellulose bonds were broken during the manufacture of the (CNC) [40].

SB presents several potential advantages as a source for CNCs. Its relatively high cellulose content, ranging from 40 to 50%, surpasses that of some other agricultural residues, offering a substantial starting material [47].

Furthermore, the cellulose microfibrils within sugarcane bagasse are reported to be less tightly bound compared to wood, which can translate to a more facile and energy-efficient CNC extraction process, ultimately leading to higher yields and reduced production costs [48, 49].

Beyond ease of extraction, CNCs derived from sugarcane bagasse often exhibit high crystallinity, a crucial attribute that significantly contributes to their reinforcing capabilities within nanocomposites, thereby enhancing mechanical properties [50].

Finally, these CNCs typically possess a favorable aspect ratio, a key morphological characteristic that facilitates effective stress transfer within composite materials, resulting in improved strength and stiffness of the final product [51].

The cost-benefit analysis of utilizing CNCs extracted from SB as a filler compared to conventional options like carbon black reveals a complex interplay of economic and performance factors. While carbon black benefits from established large-scale production and lower market prices, CNC from sugarcane bagasse offer the significant advantage of a low-cost, often waste-derived raw material, aligning with sustainable practices [52, 53].

Although current CNC production costs might be higher, ongoing research into more efficient extraction methods holds the potential for future cost reduction. From a performance perspective, CNCs have demonstrated comparable or even superior reinforcement in specific mechanical properties, alongside the added benefits of biodegradability, lower density, and potential for surface functionalization [54, 55].

**Table 3:** Crystallinity index of cellulose and CNC

Samples	Cr. I (%)	d (nm)
**Cellulose**	61.02	0.73
cellulose nanocrystals **(**CNC)	55.55	0.40

### Thermal analyses of cellulose nanocrystals (CNC)

**Fig. 4:**
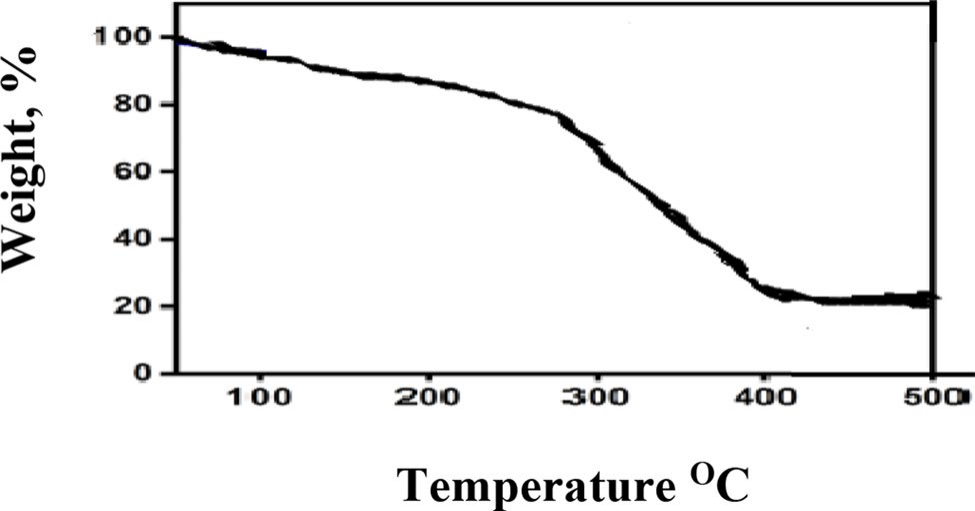
TGA of cellulose nanocrystals(CNC)

Using thermal analysis (TGA), the percentage change in weight with temperature for (CNC) from the bagasse fiber extraction method was calculated, as shown in Fig. 4. Because of the evaporation of free moisture from the nanocelluloses surface, the TGA of nanocellulose started with a temperature range of 100 to 235 °C (4.5%). Hemicellulose and lignin are removed during extraction, which results in this phenomenon. The highest level of heat stability is likewise demonstrated by nanocellulose. At temperatures higher than 235 °C, the breakdown of the fiber’s constituents, including cellulose, hemicellulose, and lignin, results in a 90% weight loss in the second stage [6, 56].

### Rheological properties

Table 4 displays the rheological properties of the EPDM mixes after they were measured for 30 min at 152 *±* 1 °C. Rheometric curves, which depict the processability and property values of the finished product, may be used to determine the ideal processing temperatures and timeframes. The rheometric characterization of EPDM at different concentrations of (CNC) shows a notable impact on the mechanical characteristics and curing behavior. Initial stiffness is indicated by the minimum torque (ML), which increases from sample E0 to E4 then decrease at E5. According to this pattern, uncured rubber’s stiffness is initially increased by (CNC), however excessive filler concentrations (as in E5).

may cause particle aggregation, which would result in a little decrease in stiffness. With (CNC), the maximum torque (MH), which indicates the final stiffness after curing, also rises these peaks in E4 at 23.92 dNm and then slightly decreases in E5 to 22.85 dNm. The idea of enhanced crosslink density and cured rubber strength using (CNC) is supported by the increase in MH. Simultaneously, the little drop in E5 indicates that too much filler may reduce crosslink effectiveness because of agglomeration. From 3.42 min in E0 to 2.05 min in E4, and then again in E5, the scorch time (ts2), a measure of the amount of time before cure starts, increases steadily. By postponing premature curing, (CNC) improve processing safety by lowering ts2; this effect seems to be limited at high concentrations (E5). Furthermore, the quantity of (CNC) typically lengthens the optimum cure time (tc90), or the amount of time required to achieve 90% of the final crosslink density. It peaks in E4 at 6.75 min and increases slightly in E5. Due to limited EPDM chain mobility, this suggests that higher quantities of cellulose nanocrystals (CNC) impede curing. This suggests an ideal range where (CNC) enhance performance without unduly extending cure time. The curing rate is measured by the cure rate index (CRI), which increases from 12.95 min⁻¹ in E0 to 21.27 min⁻¹ in E4 before decreasing to 18.18 min⁻¹ in E5. An ideal cure rate with balanced crosslinking and speed is suggested by this peak in CRI at E4. Extra (CNC) probably compromises curing effectiveness. According to the data, EPDM rheometric properties are improved by (CNC) up to an ideal concentration. Sample E4 offers the best combination of high stiffness (MH) and efficient curing (CRI), making it the most well-balanced formulation in terms of both mechanical and curing properties. So the maximum torque rises with increasing natural filler concentration while scorch and cure time decrease, are consistent with our reported results [30, 57].

**Table 4:** Rheological properties of the prepared EPDM vulcanizates

Properties	E0	E1	E2	E3	E4	E5
Minimum torque (ML), dN_m_	1.4	1.52	1.74	2.6	2.74	2.68
Maximum torque (MH), dNm	16.16	17.6	19.6	20.44	23.92	22.85
Optimium Cure Time (t_C_90), min	11.14	10.3	8.72	7.43	6.75	7.73
Scorch time t_S_2, min	3.42	2.9	2.78	2.68	2.05	2.23
Cure rate index, (CRI, min^−^1)	12.95	13.51	16.84	21.05	21.27	18.18

Where dNm: Deci Newton Meter.

### Fourier transform infrared spectroscopy for EPDM and EPDM/ (CNC)

**Fig. 5:**
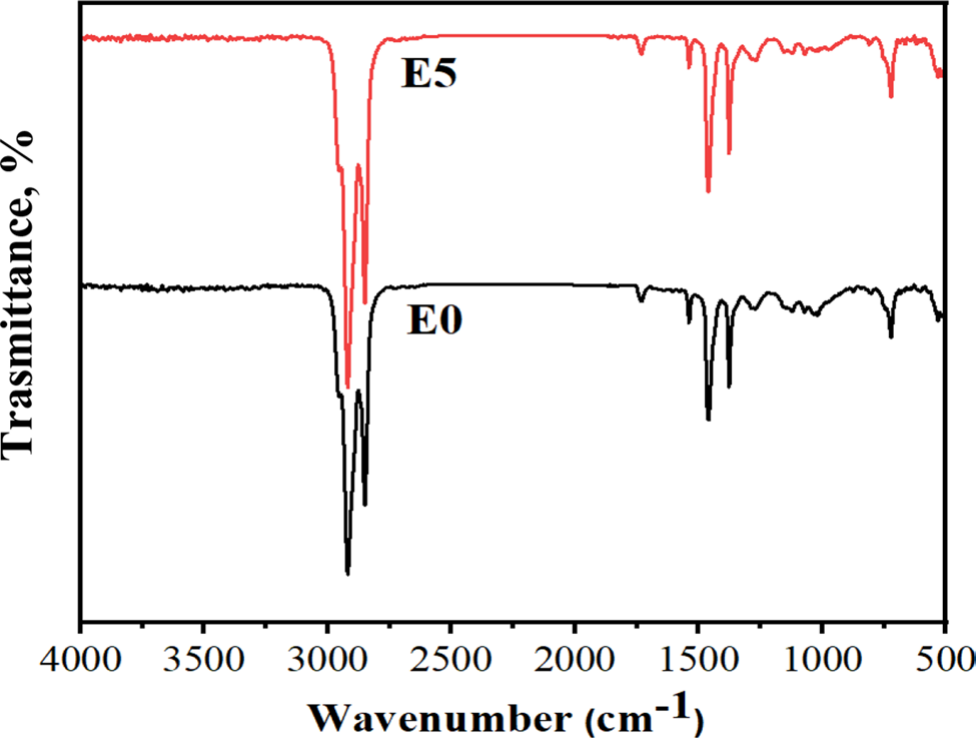
FTIR of EPDM (E0) and EPDM/ (CNC) (E5, 10phr)

The EPDM (E0) and EPDM/ (CNC) **(**E5, 10phr) as shown in Fig. 5 showed peaks between 2919.56 and 2919.83 cm^− 1^ & 2850.75–2850.95 cm^− 1^ are due to the stretching vibration of -CH_2_-, 1731.29–1732.10 cm^− 1^ is due to C = C, 1461.74–1461.88 cm^− 1^ & 1376.61–1376.58 cm^− 1^ are assigned to the in-plane bending vibration of C-H bond, and the peak between 532.00 and 532.07 cm^− 1^ is ascribed to the outplane bending vibration of C-H bond [58, 59].

Besides, C-H symmetric vibrations were observed with a similar trend that shifted from 2919.56 cm^− 1^ for E0 to a higher wavenumber at 2919.83 cm^− 1^ after adding the CNCs. It seems that the MCC influenced the chemical structure of the EPDM rubber matrix. While hydroxyl groups (OH) are typically observed around 3300 cm⁻¹, they were not distinctly present in the spectra of this MCC sample. The chemical structure of the EPDM rubber matrix was only slightly affected by the incorporation of MCC, as evidenced by minor changes, mainly in the CH asymmetric and symmetric vibrations [58].

### Impact of the concentration of cellulose nanocrystals (CNC) on mechanical characteristics of EPDM vulcanizates

The EPDM rubber Shore A hardness, modulus at 100 and 200%, elongation at break, and tensile strength are all increased in the presence of (CNC) as shown in Table 5. The tensile strength values were increased by increasing the concentration of (CNC) till 8phr and decreased after 10phr. Mechanical limitations above 10 phr caused the EPDM matrix to become immobile and interact physically, which was the reason for the drop according to some agglomeration of (CNC). As anticipated, the tensile strength of EPDM/ (CNC) was 2.82: 10.82 MPa [60, 61], which is higher than the tensile strength of EPDM vulcanizates without NCCs, which is 2.68 MPa. (CNC) essentially strengthens the prepared composite. Consequently, the more (CNC) there are in the composites, the higher their tensile strength. It is clear that up to 8 phr, the elongation at break percentage progressively increases as the concentration of (CNC) filler increases, after which it declines (see Table 5). This may be because the vulcanizates are becoming more rigid. As shown in Table 5(b), the modulus gradually increases overall for elongations of 50, 100, and 200%. The enhanced filler–matrix bonding may have improved the efficiency of stress transfer from the matrix to the filler phases [62, 63]. Compared to the EPDM matrix, the modulus 200% increased as the amount of (CNC) increased throughout the introduction process. The mechanical properties of the resulting rubber vulcanizates are greatly influenced by a number of parameters, including the size of the filler, the degree of dispersion, and the interfacial adhesion between (CNC) and EPDM chains. Adhesion and dispersion are influenced by the fillers surface modification, polarity, and processing procedure. When hydroxyl groups on the surface of (CNC) interacted with EPDM chains, hydrogen bonds were formed, which made this possible. As a result, the modulus increased (0.964, 1.27, and 1.59 MPa at 50, 100, and 200%, respectively), as well as a corresponding increase in elongation at break and tensile strength [25, 30].

**Table 5:** Mechanical properties of EPDM vulcanizates loaded with different concentrations of cellulose nanocrystals (CNC)

Samples	Tensile Strength (MPa)	Elongation at break (%)	Modulus, 50% (MPa)	Modulus, 100% (MPa)	Modulus, 200% (MPa)	Hardness, Shore A
E0	2.680 ± 0.4	245 ± 15	0.681 ± 0.3	0.94 ± 0.4	1.225 ± 0.3	60 ± 0.76
E1	2.824 ± 0.2	250 ± 13	0.696 ± 0.4	0.986 ± 0.3	1.233 ± 0.4	62 ± 0.29
E2	5.103 ± 0.1	365 ± 14	0.759 ± 0.3	1.035 ± 0.2	1.39 ± 0.4	65 ± 0.58
E3	8.440 ± 0.3	505 ± 12	0.827 ± 0.3	0.997 ± 0.3	1.302 ± 0.3	69 ± 0.35
E4	10.82 ± 0.2	572 ± 12	0.964 ± 0.1	1.27 ± 0.2	1.59 ± 0.3	72 ± 0.28
E5	7.72 ± 0.1	495 ± 13	0.741 ± 0.2	0.979 ± 0.2	1.223 ± 0.3	70 ± 0.54

### Hardness test

EPDM and EPDM/ (CNC) hardness values are listed in Table 5. In NBR vulcanizates, It has been found that the hardness values rise from 60 to 72 phr of (CNC) loading. The reinforcement effect of (CNC) in the EPDM matrix, which produces stiffness and rigidity, is the cause of the increased hardness [64–66]. This behavior suggests poor dispersion due to agglomeration at high filler content, which leads to poor interaction between the filler and the polymer EPDM rubber matrix and eventually causes the filler to lump, creating a weaker composite with low hardness, even though higher filler content also results in a decrease in hardness values [25].

### Equilibrium swelling properties

**Fig. 6:**
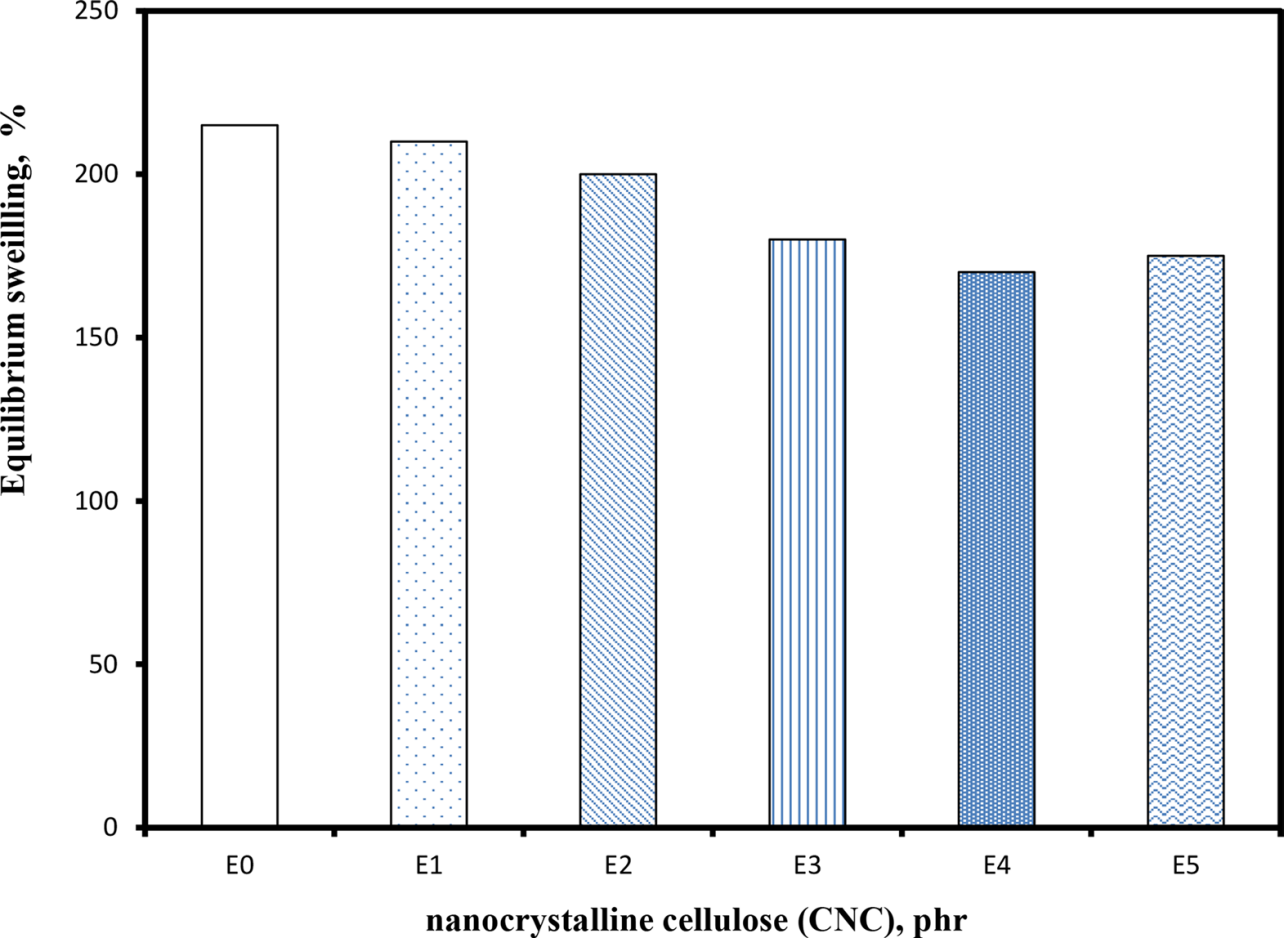
Impact of loading on equilibrium swelling of EPDM vulcanizates by cellulose nanocrystals (CNC)

Figure 6 illustrates how larger concentrations of (CNC) affect mass swell (in toluene) for EPDM composites. The equilibrium swelling in toluene clearly reduces as the amount of (CNC) increases, as seen in Fig. 6. It is clear that the weight fraction of the rubber component falls as the loading of nonswellable fillers rises. Consequently, as the loading of (CNC) increases, the equilibrium swelling of the associated vulcanizates is decreased. The swelling behavior and crosslink density more directly with mechanical property trends [30, 67].

### Crosslink density

The crosslink density (ν) and number average molecular weight between crosslinks (Mc) are calculated using swelling technique in accordance with Flory-Rehner equation. Due to the formation of hydrogen bonding in vulcanized rubber as physical bond between EPDM chains and hydroxyl group (-OH) in (CNC), which predicts the stiffness and swelling properties of rubber, number average molecular weight is a suitable indicator to predict the development of cross-linkages. The swelling properties (Mc, and ν) values of EPDM composites in toluene are measured and tabulated in Table 6. The equilibrium swelling Q and molecular weight between the crosslink points Mc values decrease with increasing the (CNC) concentration till to 8phr. On the other hand, the crosslinking density ν of the nanocomposites are increased with increasing (CNC) content up to 10phr.The strong interaction between the rubber chains may cause the formation of strong physical interactions between EPDM and (CNC) leading to increasing in the crosslink density. Also, increasing (CNC) content leads to decrease the movement of the rubber chains molecules and makes it difficult for toluene to penetrate through the rubber matrix. Therefore, At higher (CNC) concentration (> 8 phr), filler agglomeration likely caused stress concentration points, reducing tensile strength and elongation [68, 69].

**Table 6:** Swelling properties of EPDM vulcanizates loaded with different concentrations of cellulose nanocrystals (CNC)

Samples	VR	ν	Mc
E0	0.00462963	8.23343 × 10^− 9^	60728024.74
E1	0.00473934	8.59037 × 10^− 9^	58204693.87
E2	0.00497512	9.38249 × 10^− 9^	53290739.84
E3	0.00552486	1.13624 × 10^− 8^	44004945.75
E4	0.00584795	1.26135 × 10^− 8^	39640098.01
E5	0.00568182	1.1962 × 10^− 8^	41798962.59

### Scanning electron microscopy (SEM)

**Fig. 7:**
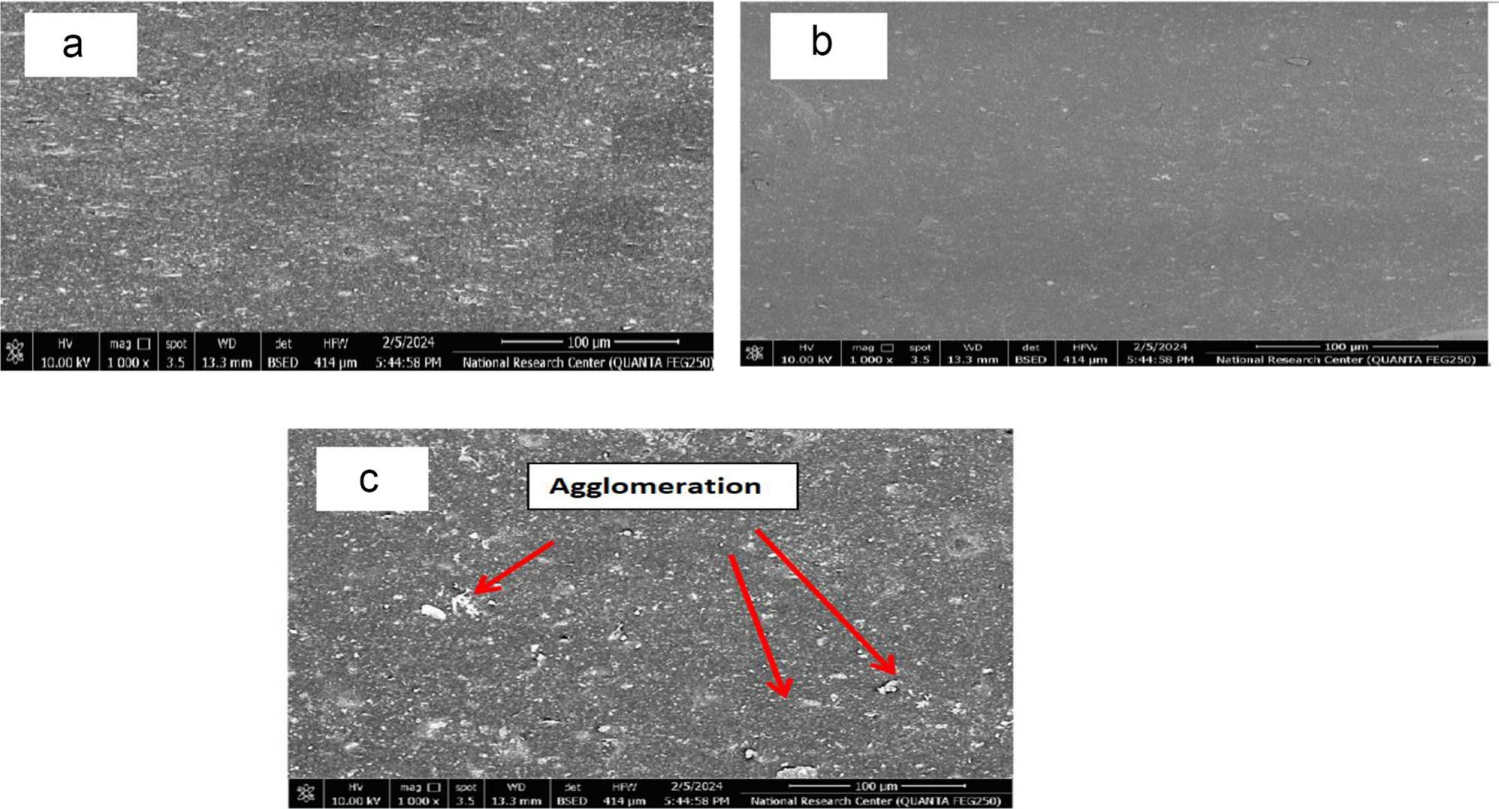
SEM. (**a**) EPDM, (**b**) EPDM/ cellulose nanocrystals (CNC) (8 phr) and (**c**) EPDM/ cellulose nanocrystals (CNC) (10 phr)

The SEM images in Fig. 7 (a, b and c) shows important information about how the morphology of EPDM polymer changes when different amounts of (CNC) are added. The samples surfaces are comparatively smooth and uniform surface, Fig. 7a, which depicts EPDM without (CNC), exhibits a typical EPDM morphology with little reinforcement. A homogeneous distribution of the (CNC) particles is visible in Fig. 7b, which depicts the ideal sample with 8 phr of NCs. Enhancing interfacial bonding and strengthening the EPDM matrix, this uniform dispersion enhances the mechanical properties of the composite. The (CNC) particles exhibit a discernible aggregation in Fig. 7c, which depicts the EPDM containing 10 phr of (CNC), which show not uniform dispersion of (CNC) and some aggregation was noted. This clustering may result in weak spots in the EPDM matrix, which would impair mechanical performance and load transfer. Because of the increased viscosity at this greater filler loading, effective dispersion during processing is probably hampered, which compromises structural integrity. The importance of maximizing the amount of (CNC) in the polymer matrix to successfully balance reinforcement and homogeneity eventually affects the mechanical characteristics and possible uses of the material [30, 63].

### Thermogravimetric data (TGA)

**Fig. 8:**
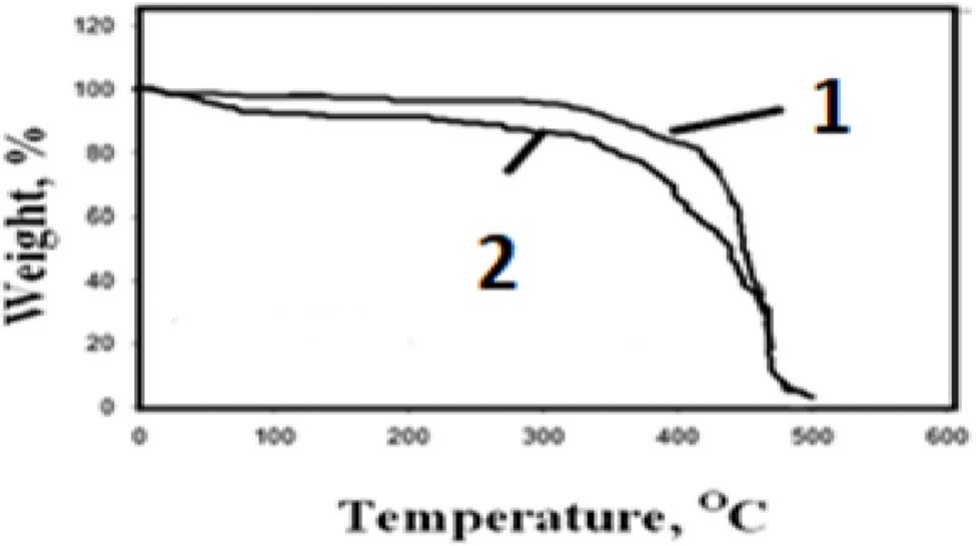
TGA curves of: 1 -EPDM, 2- EPDM/ 8 phr cellulose nanocrystals (CNC)

EPDM and EPDM/ 8 phr (CNC) vulcanizates have TGA recordings, as seen in Fig. 8 (1 and 2), respectively. The EPDM used alone shows maximum degradation temperature at 460 °C, which is higher than EPDM contained 8 phr (CNC) was used, the degradation temperature at 470 °C. Thus, the (CNC) improved the nanocomposites final products binding strength, this may be due to some hydrogen bonding formation and consequently thermal stability occur. The presence of a (CNC) was responsible for the enhanced bond strength and thermal stability of the final nanocomposite, which in turn increased the modified nanocomposite’s thermal stability (Fig. 8) [6].

## Conclusions

The impact of the (CNC), produced by hydrolyzing bleached bagasse pulp from agricultural waste with sulfuric acid, on the EPDM matrix is investigated. The yield of (CNC) has been verified by FTIR spectra, cellulose and CNCs in order to ascertain their empirical crystallinity (LOI) and average hydrogen bond strength (MHBS). The breakdown of cellulose into CNCs was demonstrated by the finding of the values of MHBS and LOI of (CNC) are lower than CNC. The prepared (CNC) had sizes ranging from 18.92 to 23.69 nm, according to TEM examination. According to TGA and XRD analysis, (CNC) have the maximum crystallinity index of 55.55% and are thermally stable. The approaches used to obtain the correct properties of (CNC) are successful. It is recommended to use (CNC) as filler for EPDM rubber at concentration at 8phr. So the strong hydrogen bonding or strong interactions and uniform dispersion between 8phr cellulose nanocrystals and EPDM matrix had observed. Overall, employing the suggested methodology to create (CNC) from sugarcane bagasse waste is a more effective and sustainable method. The described method for CNC synthesis from sugarcane bagasse, while effective at a lab scale, presents several scalability challenges, particularly concerning the use and recovery of sulfuric acid. The large volume of concentrated sulfuric acid (200 mL for 20 g pulp) poses significant handling and safety concerns at industrial levels. The subsequent neutralization through extensive dialysis and washing generates substantial volumes of wastewater, increasing treatment costs and environmental impact. While not explicitly stated, the energy consumption for heating the reaction, prolonged dialysis, repeated centrifugation, and the energy-intensive freeze-drying process would be considerable at larger scales. Efficient and cost-effective recovery and recycling of the sulfuric acid are crucial for economic viability and environmental sustainability. Furthermore, ensuring consistent quality and yield of CNCs from variable batches of sugarcane bagasse at larger volumes and optimizing the downstream processing steps (sonication, freeze-drying) for continuous operation are additional hurdles that need to be addressed for successful industrial-scale production. It is interesting to recommend that addition of 8phr (CNC) produced from sugarcane bagasse waste as filler for EPDM rubber enhanced all product properties like rheological, physico mechanical and chemical properties as well as thermal stability. Therefore, it had good dispersion through EPDM matrix as illustrated from SEM.

## Data Availability

No datasets were generated or analysed during the current study.
